# Syllable-rate-adjusted-modulation (SRAM) predicts clear and conversational speech intelligibility

**DOI:** 10.3389/fnhum.2024.1324027

**Published:** 2024-02-12

**Authors:** Ye Yang, Fan-Gang Zeng

**Affiliations:** ^1^Department of Biomedical Engineering, University of California, Irvine, Irvine, CA, United States; ^2^Department of Otolaryngology-Head and Neck Surgery, University of California, Irvine, Irvine, CA, United States

**Keywords:** objective metric, speech intelligibility, clear speech, temporal modulation, automatic speech recognition

## Abstract

**Introduction:**

Objectively predicting speech intelligibility is important in both telecommunication and human-machine interaction systems. The classic method relies on signal-to-noise ratios (SNR) to successfully predict speech intelligibility. One exception is clear speech, in which a talker intentionally articulates as if speaking to someone who has hearing loss or is from a different language background. As a result, at the same SNR, clear speech produces higher intelligibility than conversational speech. Despite numerous efforts, no objective metric can successfully predict the clear speech benefit at the sentence level.

**Methods:**

We proposed a Syllable-Rate-Adjusted-Modulation (SRAM) index to predict the intelligibility of clear and conversational speech. The SRAM used as short as 1 s speech and estimated its modulation power above the syllable rate. We compared SRAM with three reference metrics: envelope-regression-based speech transmission index (ER-STI), hearing-aid speech perception index version 2 (HASPI-v2) and short-time objective intelligibility (STOI), and five automatic speech recognition systems: Amazon Transcribe, Microsoft Azure Speech-To-Text, Google Speech-To-Text, wav2vec2 and Whisper.

**Results:**

SRAM outperformed the three reference metrics (ER-STI, HASPI-v2 and STOI) and the five automatic speech recognition systems. Additionally, we demonstrated the important role of syllable rate in predicting speech intelligibility by comparing SRAM with the total modulation power (TMP) that was not adjusted by the syllable rate.

**Discussion:**

SRAM can potentially help understand the characteristics of clear speech, screen speech materials with high intelligibility, and convert conversational speech into clear speech.

## 1 Introduction

Automatic speech recognition and synthesis are important parts of a socially intelligent human-machine interaction system. To design a well-behaved and well-performed system, one needs to predict speech intelligibility not only in quiet but also in noise and other challenging situations including different talkers and different speech styles. Indeed, speech intelligibility prediction has been a cornerstone in telecommunication. One hundred years ago, engineers were able to predict speech intelligibility based on signal-to-noise ratios (SNR) from several frequency bands (French and Steinberg, 1947; Kryter, 1962). This SNR-based prediction not only established the foundation for telephony but was also applied to a wide range of hearing and speech issues, including prediction of hearing aid and cochlear implant performance (Amlani et al., 2002).

Interestingly, the SNR-based prediction has failed for a particular style called clear speech, which produced higher intelligibility than conversational speech at the same SNR. Clear speech was first systematically studied by Picheny et al. (1985, 1986, 1989) at MIT. The clear speech benefit has been attributed to both global and phoneme-level acoustic characteristics in the clear speech, including slower speech rates (Picheny et al., 1989; Uchanski et al., 1996; Krause and Braida, 2002), greater power at high frequencies (Krause and Braida, 2004), deeper temporal modulation at low modulation frequencies (Krause and Braida, 2004; Liu et al., 2004), and more vowel-consonant contrasts (Picheny et al., 1986; Krause and Braida, 2004; Ferguson and Kewley-Port, 2007). Clear speech improves speech perception not only in noisy environments for listeners with normal hearing (Payton et al., 1994; Liu et al., 2004), but more importantly for listeners with hearing loss (Picheny et al., 1985; Ferguson and Kewley-Port, 2002; Krause and Braida, 2002; Liu et al., 2004; Smiljanić and Bradlow, 2005; Zeng and Liu, 2006).

At present, there still lacks an objective metric to characterize clear speech and predict its speech intelligibility benefit over conversational speech. Previous studies used the traditional SNR-based method or articulation index (Kryter, 1962), speech intelligibility index (ANSI-S3.5, 1997), traditional and speech-based speech transmission index (STI) (Payton and Braida, 1999). Particularly, the speech-based STI obtained good results, but it was based on modulation power spectrum estimation with a 16 s time window. The long-duration window requirement has limited its utility in predicting sentence-level intelligibility. To overcome this limitation, an envelope-regression-based STI procedure (ER-STI) was developed to predict the intelligibility of short-duration clear speech (Payton and Shrestha, 2013). However, the ER-STI not only was tested with one noise condition at −1.8 dB SNR but also predicted a clear speech benefit that was less than the human performance.

Here we proposed a novel short-time objective intelligibility metric that used speech rate adjusted modulation (SRAM) power to predict the speech intelligibility under multiple SNR levels. It can correctly predict the intelligibility of both clear and conversational speech as well as the clear speech benefit. Similar to the speech-based STI, the SRAM first estimates the modulation power spectrum of the speech envelope, but it uses time window of 1 s instead of 16 s. The most important difference between the two metrics was that the SRAM removed spectrum components below the syllable rate for intelligibility prediction, whereas the speech-based STI included all components from 0.25 to 25 Hz.

We also tested the utility of SRAM in predicting the clear speech advantage against three objective metrics: ER-STI, the hearing-aid speech perception index version 2 (HASPI-v2) (Kates and Arehart, 2021), and the short-time objective intelligibility (STOI) (Taal et al., 2011). In addition to ER-STI, we chose HASPI-v2 and STOI because of their general success in predicting speech intelligibility (Van Kuyk et al., 2018). To our knowledge, neither HASPI-v2 nor STOI has been applied to predicting the clear speech benefit.

Recent advances in automatic speech recognition (ASR) have made it a viable alternative for predicting speech intelligibility (Feng and Chen, 2022; Karbasi and Kolossa, 2022). We are not aware of the application of ASR to predict clear speech benefit. Here we also tested the speech intelligibility prediction performance of five modern ASR systems and compared them with SRAM.

## 2 Materials and **methods**

### 2.1 Human performance and model evaluation

#### 2.1.1 Speech material and subjective data

The speech material used for evaluation consisted of 144 sentences recorded from two talkers (one female, one male) in both clear and conversational styles (72 sentences in each style). These sentences were selected from Bamford-Kowal-Bench (BKB) sentences (Bench et al., 1979). The sample rate was 16,000 Hz. The silence periods before and after the speech were removed. In noise conditions, the sentences were mixed with a long-term speech-spectrum-shaped noise to produce six SNRs: −15, −10, −8, −5, 0, 5 dB. A total of 28 conditions were generated, including (6 noise levels + 1 quiet) * 2 speakers * 2 styles.

The subjective speech intelligibility data were collected using these 144 sentences from 11 subjects (see Experiment I in Liu et al., 2004). The average percentage of correctly recognized keywords was recorded as the subjective speech intelligibility. A two-parameter logistic function (Eq. 1) was used to relate the intelligibility data to SNRs and estimate the speech reception threshold (SRT):


(1) $$f\,\left(S\,N\,R\right)=\frac{1}{1+e^{-k\,\left(S\,N\,R-S\,R\,T\right)}}$$

where SRT is the SNR that produced a 50%-speech intelligibility score. The clear speech benefit (Eq. 2) is defined as the difference between the speech reception threshold of clear speech (*SRT*_*clear*_) and conversational speech (*SRT*_*conv*_):


(2) $$\delta_{S\,R\,T}=S\,R\,T_{c\,l\,e\,a\,r}-S\,R\,T_{c\,o\,n\,v}$$

#### 2.1.2 Transformation model and fitness

All objective metrics and ASR systems were evaluated using all sentences under all conditions. To compare the results between different methods, a nonlinear transformation is commonly used to convert an objective metric into a subjective speech intelligibility score (Taal et al., 2011). A four-parameter logistic model was used to transform a value (x) from objective metrics or ASR systems into a speech intelligibility score, f(x):


(3) $$f\,\left(x\right)=d-\frac{d-a}{1+{\left(x/c\right)}^{b}}$$

where *a* and *d* are the minimum and maximum values, *b* is a parameter related to the slope of the curve, and *c* is the halfway point on the curve between *a* and *d*.

The transformation model (Eq. 3) was evaluated by the normalized root-mean-square error (NRMSE) or σ (Eq. 4) between the transformed objective metric values and speech intelligibility score:


(4) $$\sigma =\frac{\sqrt{\frac{1}{S}\,\sum_{i=1}^{S}{\left(s_{i}-f\,\left(x_{i}\right)\right)}^{2}}}{s_{m\,a\,x}-s_{m\,i\,n}}$$

where *S* was the total number of conditions, *s_i_* was the averaged human intelligibility score under condition *i*, *x_i_* was the averaged objective metric value across 72 sentences under the same condition *i*, *s*_*max*_ and *s*_*min*_ were the maximum and minimum value of the human speech intelligibility, respectively. Table 1 lists the fitted parameters for all objective metrics and ASR systems.

**TABLE 1:** Fitted nonlinear transformation parameters relating subjective speech intelligibility to objective metrics.

Metric	a	b	c	d	ASR	a	b	c	d
TMP	−0.19	1.89	0.11	0.99	Amazon	0.02	0.57	0.57	1.67
SRAM	−0.21	2.22	0.08	0.99	Microsoft	0.01	1.03	1.05	2.00
SRAM_*est*_	−0.20	2.21	0.08	0.99	Google	0.07	0.50	1.16	2.00
STOI	−0.09	8.01	0.62	1.01	wav2vec2	0.10	0.41	1.37	2.00
ER-STI	0.01	4.49	0.34	0.99	Whisper	0.06	2.11	1.06	2.00
HASPI-v2	0.08	4.76	1.06	2.00					

The The four-parameter logistic model (Eq. 3) is used: $f\,\left(x\right)=d-\frac{d-a}{1+{\left(x/c\right)}^{b}}$.

### 2.2 Syllable-rate-adjusted-modulation

**FIGURE 1:**
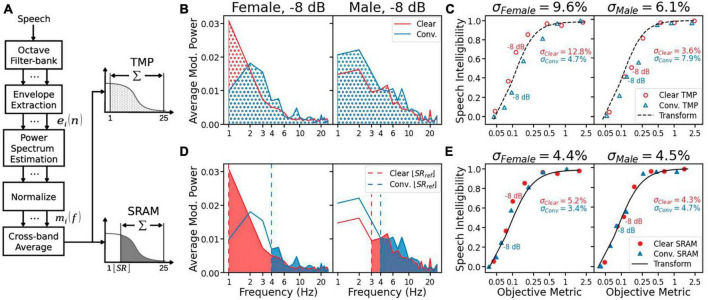
**(A)** The diagram for the total modulation power (TMP) and syllable-rate-adjusted-modulation (SRAM). The processing is the same between TMP and SRAM, except for the last stage in which the modulation power is removed below the syllable rate for SRAM (Eqs 7, 8). **(B)** The averaged modulation power spectrum for the sentence “The orange was very sweet” at –8 dB SNR, produced in either clear (red line) or conversational (blue line) speech style by a female (left panel) and male (right panel) speaker. The shaded area under each line equals the TMP in its corresponding condition. **(C)** Speech intelligibility as a function of TMP. The speech intelligibility data are represented as red circles for clear speech and blue triangles for conversational speech. The black line represents the best fitted nonlinear transformation (Eq. 3), with the overall error (σ) being displayed on the top of the panel, and the clear and conversational speech errors inside the panel. **(D)** Same as **(B)** for SRAM, except that the modulation power is removed below the syllable rate (vertical dashed lines: red = clear speech and blue = conversational speech). **(E)** Same as **(C)** for SRAM, except for the solid symbols.


Figure 1A illustrates the algorithms for calculating total modulation power (TMP) and SRAM. The front-end processing is the same. First, the speech was filtered into seven bands, using six sixth-order octave-wide Butterworth filters with center frequencies from 125 Hz to 4 kHz, and additionally a sixth-order Butterworth 6-kHz high-pass filter. For each band, the intensity envelope *e*_*i*_(*n*) (Eq. 5) was extracted from the filtered signal *s*_*i*_(*n*) using Hilbert transform *H*:

(5) $$e_{i}\,\left(n\right)=\sqrt{s_{i}\,{\left(n\right)}^{2}+H\,{\left\{s_{i}\,\left(n\right)\right\}}^{2}}$$

where *n* = 1, 2, … , *N*, with *N* being the total length of the speech envelope.

The modulation power spectrum in each band *P*_*i*_(*f*) was estimated from the intensity envelope using Bartlett’s method (Bartlett, 1948) with a 1 s window, which yielded a 1 Hz frequency resolution. The resulting modulation power spectrum *P*_*i*_(*f*) was normalized by the average power of the envelope and the window duration *D*:


(6) $$m_{i}\,\left(f\right)=\frac{P_{i}\,\left(f\right)}{\frac{D}{N}\,\sum_{n=1}^{N}e_{i}\,{\left(n\right)}^{2}}$$

Total modulation power (TMP) and SRAM differed in the final stage. The TMP was the result of summing up band-averaged *m*_*i*_(*f*) (Eq. 6) from 1 Hz to 25 Hz:


(7) $$\text{TMP}=\sum_{k=1}^{25}\frac{1}{7}\,\sum_{i=1}^{7}m_{i}\,\left(k\right)$$

Syllable-rate-adjusted-modulation (SRAM), in contrast, was the result of summing up band-averaged *m*_*i*_(*f*) (Eq. 6) from integral part of the syllable rate (SR) to 25 Hz:


(8) $$\text{SRAM}=\sum_{k=\left\lfloor S\,R\right\rfloor }^{25}\frac{1}{7}\,\sum_{i=1}^{7}m_{i}\,\left(k\right)$$

Figure 1B gives an example of the TMP calculation for the female speaker (left panel) and the male speaker (right panel) in either the clear (red) or conversational (blue) style at −8 dB SNR. As indicated by the shaded area, the TMP was relatively similar between clear and conversational speech, making it difficult to predict the clear speech benefit. This difficulty was also seen for the TMP averaged over all sentences, as shown in Figure 1C (left panel, female): the objective metrics were close to 0.1 on the *x*-axis for both the clear and conversational speech at −8 dB SNR, but their corresponding subjective speech intelligibility scores were vastly different at 0.7 and 0.2, respectively. As a result, the transformation model (black dashed line in Figure 1C) underpredicted the clear speech intelligibility while overpredicted the conversational speech intelligibility, giving rise to a relatively large error for the female speaker (σ_*Female*_ = 9.6%).

Figure 1D shows the effect of syllable rate on SRAM. It removed the power for modulation frequencies below the syllable rate, which decreased the modulation power more for conversational speech than clear speech. In particular, the SRAM and TMP were the same for female clear speech, because it had a syllable rate close to 1 Hz. The net effect was that the SRAM (Figure 1E) produced much smaller errors than the TMP.

### 2.3 Syllable rate

The calculation of SRAM requires prior knowledge of the speech syllable rate. We used two methods (detailed in sections “2.3.1 Syllable rate reference” and “2.3.2 Syllable rate estimation”) to determine the syllable rate, depending on the availability of the speech script.

#### 2.3.1 Syllable rate reference

**FIGURE 2:**
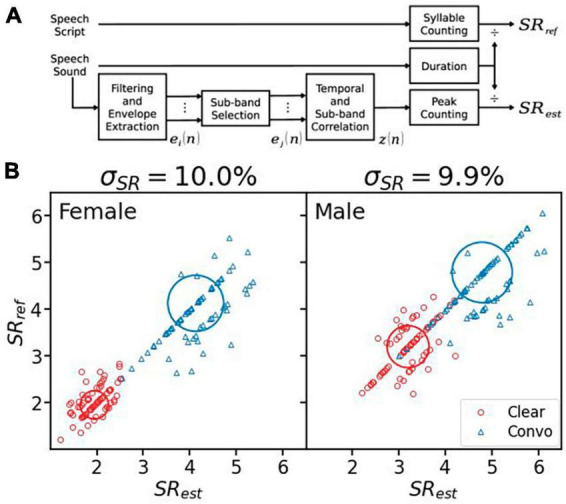
Syllable rate (SR) calculation and evaluation. **(A)** Calculating *SR*_*ref*_ from the speech script and estimating *SR*_*est*_ from the speech sound. **(B)** The reference syllable rates vs. the estimated values for the female (left panel) and the male (right panel) speaker. The clear speech data are represented by red circles, while the conversational speech by blue triangles. The center of thick circles represents the averaged syllable rates, and the radius represents one standard deviation. The error (σ_*SR*_) is displayed on the top of each panel.

If the speech script is available, the gold standard for syllable rate calculation is simply taking the ratio of the syllable count of the script over the total speech duration in seconds (upper path in Figure 2A). Here the syllable count was obtained from the speech script using the Carnegie Mellon University Pronouncing Dictionary (CMUdict).^1^ We first translated each word in the sentence into ARPAbet notations, then counted each vowel notation as one syllable.

#### 2.3.2 Syllable rate estimation

If the speech script is not available, the syllable rate can be estimated from the clean speech sound (lower path in Figure 2A). The overall estimation method was similar to Wang and Narayanan (2007). First, the speech was filtered by the same seven filters as described in section “2.2 Syllable-rate-adjusted-modulation.” Band-specific envelopes were extracted using Hilbert transformation (Eq. 5), then down-sampled to 100 Hz and low-passed by a second-order Butterworth filter with a 25 Hz cutoff frequency. Second, temporal (Eq. 9) and sub-band (Eq. 10) correlations were calculated from *M* out of seven filtered envelopes with the highest energy ($e_{j}^{\prime }\,\left(n\right),j=1,2,\ldots ,M$). In this study, M was set to 4. Different from Wang and Narayanan (2007), the temporal correlation was calculated as below:


(9) $$y_{j}\,\left(n\right)=\sqrt{\frac{1}{K}\,\left|\sum_{k=1}^{K}w_{k}\,e_{j}^{\prime }\,\left(n\right)\,e_{j}^{\prime }\,\left(n+l\right)\right|},l=k-\left\lfloor \frac{K}{2}\right\rfloor -1$$

where *w*_*k*_, *k* = 1, 2, … , *K*, was the Hann window with length *K* = 11 and $e_{j}^{\prime }\,\left(n+l\right)=0$ if *n* + *l* = 0 or *n* + *l* = *N*. This temporal correlation favored vowels over consonants.

Third, the sub-band correlation of the resulting temporal correlation was calculated the same as Wang and Narayanan (2007):


(10) $$z\,\left(n\right)=\sqrt{\frac{2}{M\,\left(M-1\right)}\,\sum_{j=1}^{M-1}\sum_{l=j+1}^{M}y_{j}\,\left(n\right)\,y_{l}\,\left(n\right)}$$

*z*(*n*) was then subject to min-max normalization. Peaks in the normalized correlation with predominance (*p*) greater than 0.07 were counted as syllables. The syllable rate was estimated as the number of peaks divided by the speech duration.

The SRAM implementation including the syllable rate estimation algorithm is available at https://github.com/yangye1098/SRAM.git.

#### 2.3.3 Syllable rate evaluation

The performance of the syllable rate estimation was also measured by the NRMSE or σ_*SR*_ (Eq. 11) between the reference syllable rate and the estimated syllable rate:


(11) $$\sigma_{S\,R}=\frac{\sqrt{\frac{1}{S}\,\sum_{i=1}^{S}{\left(S\,R_{r\,e\,f,i}-S\,R_{e\,s\,t,i}\right)}^{2}}}{S\,R_{r\,e\,f,m\,a\,x}-S\,R_{r\,e\,f,m\,i\,n}}$$

where *S* was the total number of sentences, *SR*_*ref*,*i*_ and *SR*_*est*,*i*_ were the reference syllable rate and the estimated syllable rate for the *i^th^* sentence, respectively, *SR*_*ref*,*max*_ and *SR*_*ref*,*min*_ were the maximum and minimum value of the reference syllable rate, respectively.

Figure 2B shows the performance of the syllable rate estimation algorithm. The overall error was ∼10% for female and male speakers. Of the total 288 sentences, 163 sentences (57%) were estimated to have the same syllable rate as that from the speech script. If integer precision was used due to the frequency resolution, 215 sentences (75%) produced the correct syllable rate.

### 2.4 Reference metrics

We compared SRAM with three established objective intelligibility metrics: ER-STI, HASPI-v2 and STOI. ER-STI determined the linear regression coefficients between the normalized intensity envelopes of the degraded and reference speech in seven frequency bands. These regression coefficients were then translated to an STI index. The ER-STI values were calculated using the original method to evaluate intelligibility of clear and conversational speech with the window length being the sentence duration (Payton and Shrestha, 2013).

Hearing-aid speech perception index version 2 (HASPI-v2) was based on a peripheral auditory processing model and envelope modulation analysis. HASPI-v2 extracted the speech envelope in 32 auditory frequency bands. The envelope was then filtered into the 10 modulation frequency bands, within which cross-correlation was obtained between cepstral coefficients of the degraded and reference speech. The cross-correlation values were mapped to speech intelligibility using a trained neural network (Kates and Arehart, 2021). The HASPI-v2 values were calculated using a python implementation with a 5-dB HL hearing threshold across all frequencies.^2^

Short-time objective intelligibility (STOI) calculated correlation coefficients between time-frequency representations of the degraded and reference speech (Taal et al., 2011). The STOI values were calculated using a python implementation with all default settings.^3^

### 2.5 Automatic speech recognition

We evaluated performance of five ASR systems, including three commercial cloud-based services (Amazon Transcribe, Microsoft Azure Speech-To-Text, and Google Speech-To-Text) and two recent open-source models (wav2vec2; see Baevski et al., 2020 and Whisper see Radford et al., 2022). The default settings were used for the three commercial systems. The official implementations of wav2vec2 and Whisper provided in Hugging Face were used. The pretrained model tested was “large-960h” for wav2vec2 and “large-v2” for Whisper.

## 3 Results

### 3.1 Syllable-rate-adjusted-modulation

**FIGURE 3:**
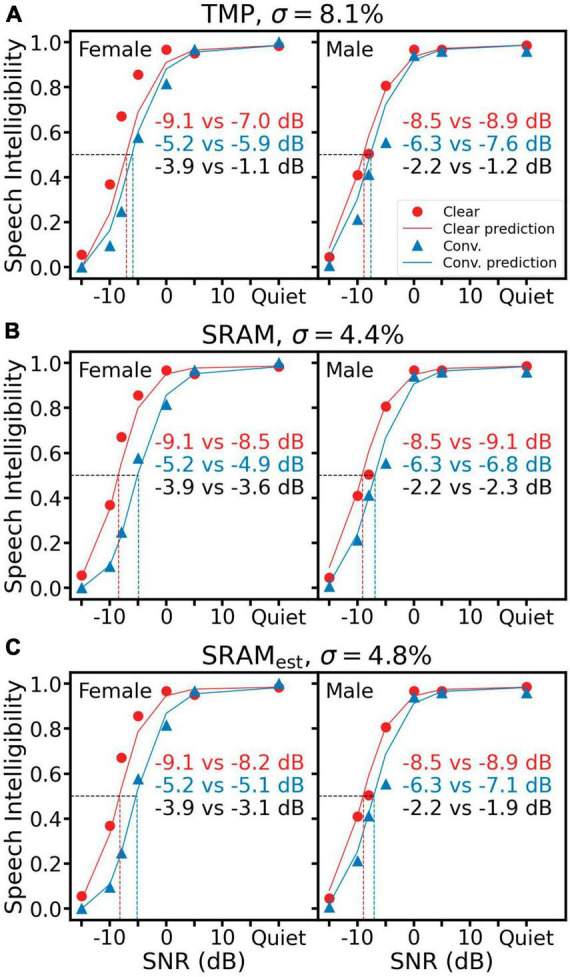
Speech intelligibility prediction using TMP **(A)**, SRAM **(B)** and SRAM_*est*_ **(C)**. Left panels show results for the female speaker and right panels for the male speaker. In each panel, the same subjective intelligibility data are represented by red circles for the clear speech and blue triangles for the conversational speech. The red solid line represents predictions for clear speech and the blue solid line for conversational speech. The prediction error (σ) is displayed on the top of each panel. Speech reception threshold or SRT is the SNR in dB, at which 0.5 speech intelligibility is achieved (the horizontal dotted line in each panel). The predicted SRT is represented as the vertical dotted red line for clear speech and the vertical dotted blue line for conversational speech. The actual vs. predicted SRT values are displayed as the red text for clear speech and the blue text for conversational speech. The clear speech benefit is the SRT difference between clear and conversational speech, with its actual vs. predicted values being displayed as the black text.

First, all panels in Figure 3 display the same human speech recognition data (red circles = clear speech; blue triangles = conversational speech). For both female (left panels) and male (right) speakers, speech intelligibility increased as a function of SNR, but the intelligibility was consistently higher for the clear speech than the conversational speech at the same low SNRs from −15 to 0 dB. This “clear speech benefit” (Eq. 2) is quantified by the difference in speech reception threshold (SRT), or the SNR at which 50% speech intelligibility is achieved (the horizontal dashed line). For the female speech, *SRT*_*clear*_ was −9.1 dB and *SRT*_*conv*_ was −5.2 dB, resulting in a clear speech benefit δ_*SRT*_ of −3.9 dB (= −9.1 to −5.2 dB, left black text). For the male speech, the corresponding values were −8.5, −6.3, and −2.2 dB, respectively.

Figure 3A shows TMP predictions (solid lines and right text in each panel, with red corresponding to clear speech and blue to conversational speech). The TMP under-predicted the clear speech benefit by 2.8 dB (= −1.1 to −3.9 dB) for the female speaker (left panel) and 1.0 dB (= −1.2 to −2.2 dB) for the male speaker (right panel). The overall prediction error (σ) was 8.1%. Figure 3B shows the SRAM prediction, which followed more closely to the human data than the TMP prediction, with a clear speech benefit difference of 0.3 dB for the female speaker and −0.1 dB for the male, and an overall σ of 4.4%. Figure 3C shows the SRAM prediction using the estimated syllable rates (SRAM_*est*_), which produced slightly worse prediction than the SRAM but still better than the TMP, with a clear speech benefit difference of 0.8 dB for the female speaker, 0.3 dB for the male and an overall σ of 4.8%.

### 3.2 Reference metrics

**FIGURE 4:**
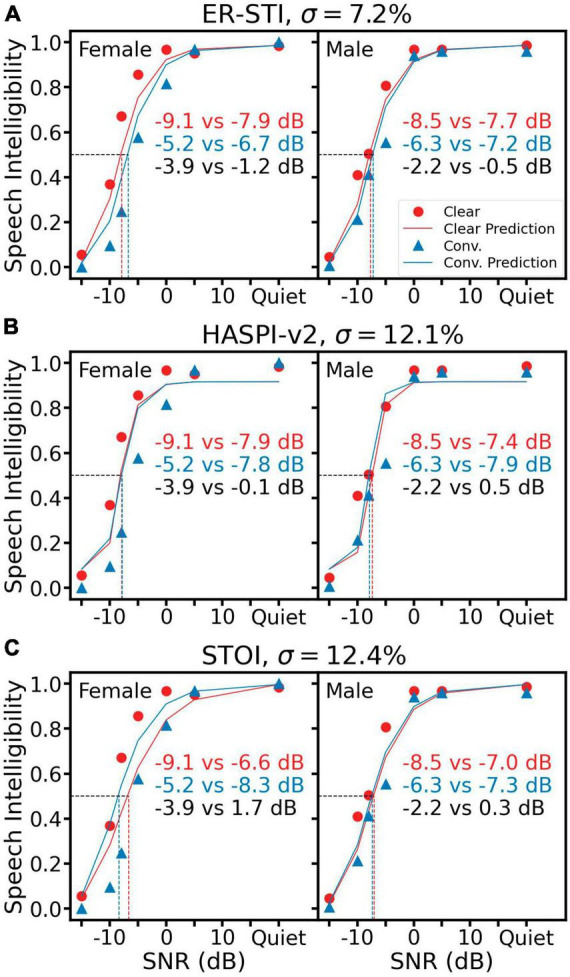
Speech intelligibility prediction using three objective reference metrics: ER-STI **(A)**, HASPI-v2 **(B)** and STOI **(C)**. All representations are the same as Figure 3.

Figure 4 shows the same human recognition data along with predictions from three reference objective metrics. Figure 4A shows that ER-STI produced an overall σ of 7.2%, which was similar to the TMP error but worse than both SRAM errors (Figure 3). On the other hand, HASPI-v2 (Figure 4B) and STOI (Figure 4C) had much higher prediction errors of 12.1 and 12.4%, respectively. Worse yet, HASPI-v2 and STOI predicted higher intelligibility for the conversational speech than the clear speech in three of the four conditions (Figure 4B male, Figure 4C both female and male), as indicated by the positive δ_*SRT*_ values in clear speech benefit.

### 3.3 Automatic recognition systems

**FIGURE 5:**
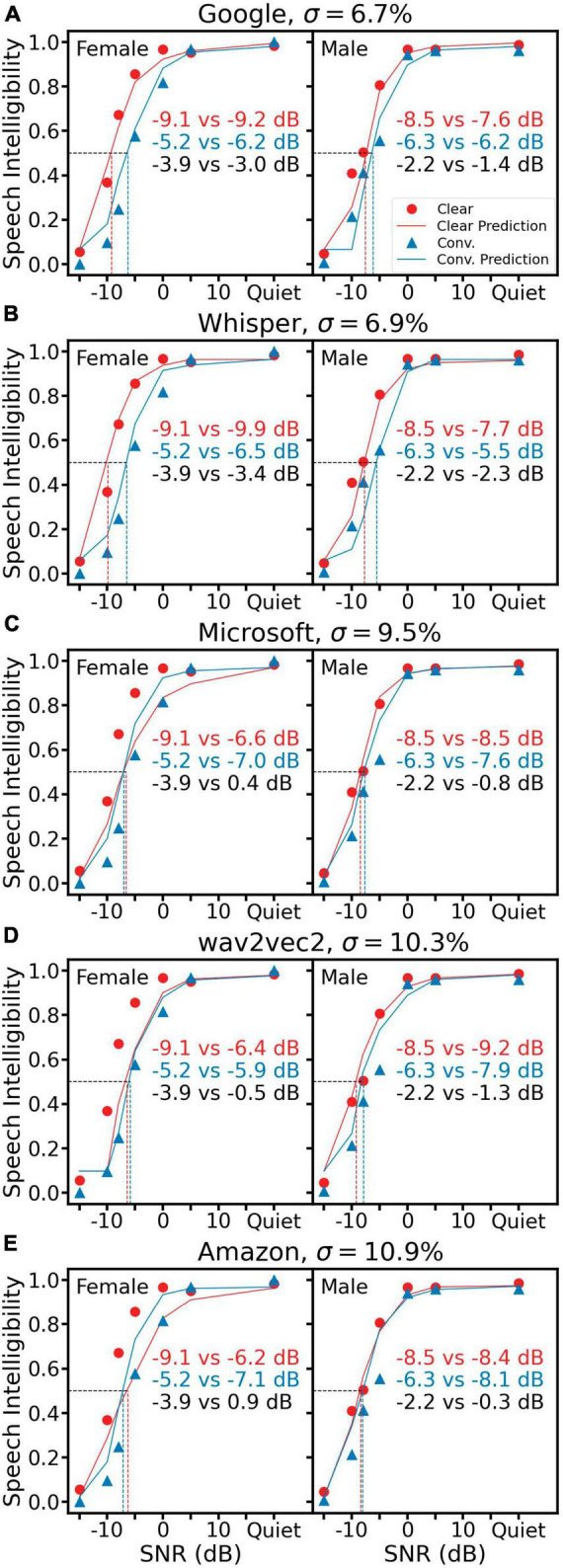
Speech intelligibility prediction using five ASR systems, Google **(A)**, Whisper **(B)**, Microsoft Azure **(C)**, wav2vec2 **(D)** and Amazon Transcribe **(E)**. All representations are the same as Figure 3.

Figure 5 shows the same human data with predictions from five ASR systems. Among them, Google (Figure 5A) and Whisper (Figure 5B) produced an overall similar prediction error of 6.7 and 6.9%, respectively, which was better than three reference metrics and the TMP but worse than the two SRAM predictions. On the other hand, the other three ASR systems produced a higher prediction error around 10%, with half conditions predicting a reversed trend in the clear speech benefit (Figure 5C female and Figure 5E both female and male).

## 4 Discussion and conclusion

In this study, we presented SRAM, an objective speech intelligibility metric based on modulation power above the syllable rate (Figure 1). We also presented an automatic syllable rate estimation algorithm in cases where speech script was not available (Figure 2). The SRAM outperformed not only the modulation power model without the syllable rate adjustment but also three acoustic-based objective metrics and five ASR systems in terms of predicting both the speech reception thresholds and the clear speech benefits (Figures 3–5). Importantly, with a one-second window, the SRAM can predict sentence-level intelligibility.

A major reason for the SRAM’s good performance is the slower syllable rate for clear speech than conversational speech. For example, the sentence “The orange was very sweet,” spoken by the female speaker, had a rate of 1.9 syllables/sec in clear style and 4.3 syllables/sec in conversational style; the same sentence, spoken by the male speaker, had a rate of 3.6 and 5.0 syllables/sec in clear and conversational style, respectively (Figure 1D, vertical dashed lines). The slower rates contributed to greater SRAM values for clear speech than conversational speech. There might be additional modulation power differences between the clear and conversational, because a talker could maintain a similar syllable rate between the two styles (Krause and Braida, 2004). Future work is needed to address this issue.

There is also evidence for humans not relying on the information at the modulation frequencies below the syllable rate to understand speech. For example, Drullman et al. (1994) found that removing components with modulation frequencies lower than 4 Hz, the average syllable rate for conversational speech, does not affect speech recognition. These below-syllable-rate components carry prosodic information such as stress rather than speech intelligibility (Goswami, 2019).

While the three reference objective metrics could predict speech intelligibility in noise, their performance was worse than SRAM in predicting the clear speech benefit. There are several possible reasons for this discrepancy. First, all three metrics were based on correlation between clean and noisy speech, which theoretically included all modulation frequencies. The inclusion of all modulation frequencies explains the similar performance between TMP and the three metrics. Second, STOI used a time-window of 384 ms to calculate the correlation, which would ignore modulation frequencies below 2.6 Hz. Because the female clear speech has a syllable rate of 1 Hz, STOI did not capture the modulation power between 1 and 2.6 Hz in the clear speech, resulting in predicting prediction a clear speech disadvantage (Figure 4C left panel). Third, HASPI-v2 predicted similar intelligibility between the clear and conversational speech at all SNRs, suggesting that the peripheral auditory model in HASPI-v2 likely extracts long-term spectral information, which was identical regardless of the speech style.

When tested with our dataset of conversational and clear speech, all five ASR systems performed relatively well in quiet, but they behaved differently in noisy conditions. Google and Whisper performed reasonably close to humans, while Microsoft Azure, wav2vec2 and Amazon could not recognize the female clear speech well. This suggests these three systems may fail to recognize speech of certain style when deployed in noisy environment. Since ASR is an important part of the human-machine interaction system, either the design or training materials or both need to be refined to achieve realistic performance under challenging situations.

One potential application of SRAM is evaluation of synthesized voices. For a socially intelligent human-machine interaction such as a voice assistant, it is crucial to select a clear voice that is intelligible not only in quiet but also in background noise. Another application is to use SRAM to pre-screen training materials that fine-tune a speech synthesizer.

The present study demonstrated that a syllable-rate-adjusted-modulation index or SRAM can predict sentence-level speech intelligibility and the intelligibility benefit of clear speech over conversational speech at the same signal-to-noise ratio. Moreover, SRAM is better than existing objective metrics and current automatic speech recognition systems. Future study is needed to test the effect of SRAM on predicting speech of various styles and explain why syllable rate plays such an important role in speech intelligibility.

## Data availability statement

The original contributions presented in this study are publicly available. This data can be found here: clear speech data for syllable-rate-adjusted-modulation (SRAM) https://zenodo.org/records/8432843.

## Ethics statement

The studies involving humans were approved by the University of California, Irvine. The studies were conducted in accordance with the local legislation and institutional requirements. Written informed consent for participation was not required from the participants or the participants’ legal guardians/next of kin in accordance with the national legislation and institutional requirements.

## Author contributions

YY: Conceptualization, Formal Analysis, Investigation, Methodology, Software, Visualization, Writing – original draft, Writing – review & editing. F-GZ: Conceptualization, Funding acquisition, Supervision, Writing – review & editing.

## Footnotes

1
http://www.speech.cs.cmu.edu/cgi-bin/cmudict

2
https://github.com/claritychallenge/clarity

3
https://github.com/mpariente/pystoi
